# Indigenous people doing citizen science to assess water quality using the BMWP in rivers of an arid semi-arid biosphere reserve in Mexico

**DOI:** 10.1038/s41598-024-65903-7

**Published:** 2024-07-02

**Authors:** Eugenia López-López, Jacinto Elías Sedeño-Díaz, Axel E. Rico-Sánchez, Edgar Andres Zariñana-Andrade, Fernando Reyes-Flores, Leticia Soriana-Flores

**Affiliations:** 1 Instituto Politécnico Nacional, Escuela Nacional de Ciencias Biológicas, Laboratorio de Evaluación de la Salud de los Ecosistemas Acuáticos, Prolongación de Carpio y Plan de Ayala s/n, Col. Santo Tomás, Miguel Hidalgo, C.P. 11340 Mexico City, Mexico; 2 Instituto Politécnico Nacional, Coordinación Politécnica para la Sustentabilidad, Av. Instituto Politécnico Nacional. Esq. Wilfrido Massieu, Col. San Pedro Zacatenco, Gustavo A. Madero, C.P. 07738 Mexico City, Mexico; 3 Posgrado en Hidrociencias, Colegio de Postgraduados, Carretera México-Texcoco 36.5 km, C.P. 56264 Montecillo, Texcoco, Mexico; 4 Dirección de la Reserva de la Biósfera Tehuacán-Cuicatlán, Comisión Nacional de Áreas Naturales Protegidas, 5 de Mayo 1611, col. Aquiles Serdán, C.P. 75750 Tehuacán, Puebla Mexico

**Keywords:** Hydrology, Environmental impact

## Abstract

Arid and semi-arid areas are among the most threatened ecosystems on the planet. The Tehuacán-Cuicatlán Biosphere Reserve (TCBR), in southeastern Mexico, is an arid and semi-arid area with high biological diversity and human settlements of eight ethnic groups. Two rivers drain the reserve, Río Grande (RG) and Río Salado (RS), which are not subject to water quality monitoring by government agencies; however, measures of water quality of these rivers are needed to focus conservation actions on this resource. This work aimed to test the effectiveness of participatory water quality monitoring with the participation of three actors: Reserve management leaders, local communities, and academics, to monitoring water quality in the rivers of the TCBR. Ninety-two residents were trained to carry out water quality biomonitoring using the Biological Monitoring Working Party (BMWP) index calibrated for the reserve. The BMWP uses macroinvertebrate families to display numerical and categorical water quality scores. Additionally, the Water Quality Index (WQI) was assessed and the Normalized Difference Vegetation Index (NDVI) of the riparian zones was estimated in each study site. The mean WQI scores were 69.24 for RS (no treatment necessary for most crops and necessary treatment for public water supply) and 75.16 for RG (minor purification for crops requiring high-quality water and necessary treatment for public water supply). The BMWP showed five water quality categories (Excellent, Very Good, Good, Fair, and Poor), showing higher water quality scores in the upper portion of the basins and capable of discriminating study sites with lower scores close to human settlements. At one study site, data from participatory monitoring impelled actions taken to address a pollution source and influenced policy focus, reaching the maximum level of participatory-based monitoring. This led to avoid the discharge of wastewater into the river to conserve and protect the water resource. WQI is closely related to BMWP; however, the latter was far more sensitive to detecting areas affected by domestic water discharges. The NDVI presented low values for the TCBR, being lower in RS (the driest area). Although the NDVI showed a weak relationship with BMWP values, areas with higher NDVI values generally achieved higher BMWP values. The results of this study highlight the high sensitivity of the BMWP to detect several water quality conditions in the rivers running through the TCBR when compared to WQI. In addition, the usefulness of biomonitoring using the BMWP index was evident, as well as the importance of the participation of local inhabitants contributing to the knowledge of water quality in biosphere reserves and carrying out timely measures that allow the rivers in these reserves to be maintained in good condition.

## Introduction

Approximately 40% of the human population lives in arid and semi-arid areas (including a significant fraction of the poorest population on the planet), which also face environmental stress^1^. As a result, they have developed strategies for managing these ecosystems and hence survive under a water scarcity regime^2^. Additionally, these regions provide various ecosystem services, including food, timber resources, plants of medical or cultural importance, water regulation, and carbon sequestration^3^.

Unfortunately, arid and semi-arid areas are among the most degraded ecosystems worldwide^4^. This degradation is largely associated with soil deterioration processes^5^. Likewise, the loss of biodiversity and ecosystem services erodes cultural identity, leading to the loss of knowledge, culture, and practices that could stop and reverse the degradation of these ecosystems, jeopardizing the achievement of the objectives of the 2030 Agenda for Sustainable Development^6^.

In these ecosystems, water is important not only in terms of quantity but also in terms of quality. Interest in monitoring water quality is related to the demand for water to meet basic needs with optimal water quality according to sustainability criteria^7^. The assessment of water quality is particularly relevant since the diverse water uses demand different quality standards to achieve sustainability^7^.

Water quality includes multiple physical, chemical, and biological variables that describe environmental conditions^8^. Adequate water quality assessment requires monitoring programs that ensure the recording of quality parameters in critical periods (peak dry or rainy seasons)^9^. This information is particularly required for intermittent rivers that have received little attention, such as some rivers in the Tehuacán-Cuicatlán Biosphere Reserve (TCBR), southern Mexico, located in the southernmost arid zone of North America. The TCBR is considered the most diverse arid-semi-arid area in North America by UNESCO^10^ and coincides with a global biodiversity hotspot. The rivers running across this reserve include perennial and intermittent systems^11,12^. These rivers are the source of water for wild aquatic and terrestrial biota in one of the most diverse biosphere reserves in Mexico^13^. In addition, the TCBR gave rise to the development of Mesoamerica, one of the cradles of civilization in the world and is inhabited by native human populations from eight pre-Columbian cultures (called indigenous peoples: Cuicatecos, Chinantecos, Chocholtecos, Ixcatecos, Mazatecos, Nahuas, Mixtecos, and Popolocas) who require water to meet their needs: human consumption, crop irrigation, and livestock consumption^14^. This region is considered the area of origin of corn domestication, for which it was recognized as a *mixed property* by the World Heritage Committee of UNESCO^10^ “Tehuacán-Cuicatlán Biosphere Reserve: Originary Habitat of Mesoamerica”. Although water is involved in various ecosystem services, the TCBR does not have water quality monitoring stations as part of the National Water Commission Monitoring Network^15^, the agency responsible for monitoring water quality in México. This lack of water quality data limits the development of effective resource management strategies, particularly in remote regions (far from large cities) that require water for different uses.

On the other hand, the use of alternative monitoring methods with biological indicators (biomonitoring) allows water quality assessments at a lower cost than evaluations based on physicochemical parameters. Biomonitoring has several advantages, as, in addition to being affordable, it offers a high degree of precision^16,17^. Biomonitoring has been extensively used in different countries around the world^18^. Aquatic macroinvertebrates stand out among the groups of organisms used for biomonitoring. The advantages of aquatic macroinvertebrates include their low mobility, abundance, ubiquity, easy collection and identification at the family level, close contact with water and sediment, and differential tolerance to pollution (ranging from highly sensitive to extremely tolerant to pollution)^19^. Furthermore, the Biological Monitoring Working Party (BMWP) index is one of the indices used for water quality bioassessment in various parts of the world^18^. BMWP measures the response of macroinvertebrates to the oxygen deficit caused by organic matter breakdown. The analysis of these responses, induced by organic pollution, allows calculating sensitivity values for each macroinvertebrate group^16^. Preliminary studies in the TCBR have shown the bioindication potential of aquatic macroinvertebrates in water quality assessments^11^.

Furthermore, participatory monitoring, also known as community-based monitoring, is a social practice that links different stakeholders involved in environmental management^20^. This type of monitoring focused on the assessment of water quality allows obtaining data on environmental conditions (and thus, filling knowledge gaps about water quality) and contributes to the development of participatory societies that promote the conservation and sustainable use of natural resources and the development of sustainable management strategies for water resources^21^.

The TCBR is home to small human communities (total population of approximately 35,724 inhabitants, with a population density of 0.13 inh./km^2^)^14^ that are distant from each other and whose access requires several travel hours along winding and mountainous roads. Various streams run within the reserve that provide water to these populations. As already mentioned, the TCBR lacks water monitoring sites by official agencies. The only evaluations available regard physicochemical water quality in 12 study sites in the main streams of the TCBR, Río Grande and Río Salado, from a previous study by López-López et al.^11^. However, the local inhabitants have expressed a legitimate interest in knowing the water quality of their rivers and streams, which are tributaries of Río Salado and Río Grande. The engagement of indigenous peoples in participatory monitoring represents an opportunity to carry out water quality assessments in regions that lack water quality monitoring stations. This would increase the coverage of the monitoring area in the TCBR, filling current knowledge gaps on water quality. In addition, local communities would gain the ability to make decisions about the use of water resources.

Managing aquatic resources in the TCBR requires water quality records. This involves the need to evaluate the ecological condition of aquatic ecosystems with social participation, including spatio-temporal variations, to set a baseline of water quality conditions and focus management strategies according to the different uses of water. The present study aimed to evaluate the water quality of rivers that run through the Tehuacán-Cuicatlán Biosphere Reserve through participatory monitoring using the BMWP index calibrated for this reserve. Additionally, a geographic extension test of the BMWP index was carried out with participatory monitoring data, including the participation of TCBR managers, local (indigenous) communities, and scientists. The biomonitoring results were compared with those of the physicochemical assessment of water (through the water quality index, WQI of Dinius^22^) and the normalized difference vegetation index (NDVI), to explore the relationship of the physicochemical condition with the bioindication assessment and analyze the influence of riparian vegetation on the physicochemical and biological water quality. Finally, we discuss the advantages of participatory monitoring with the joint participation of various stakeholders to obtain reliable information on water quality that can inform sustainable management plans for water resources for the TCBR.

## Methods

### Study area

**Figure 1:**
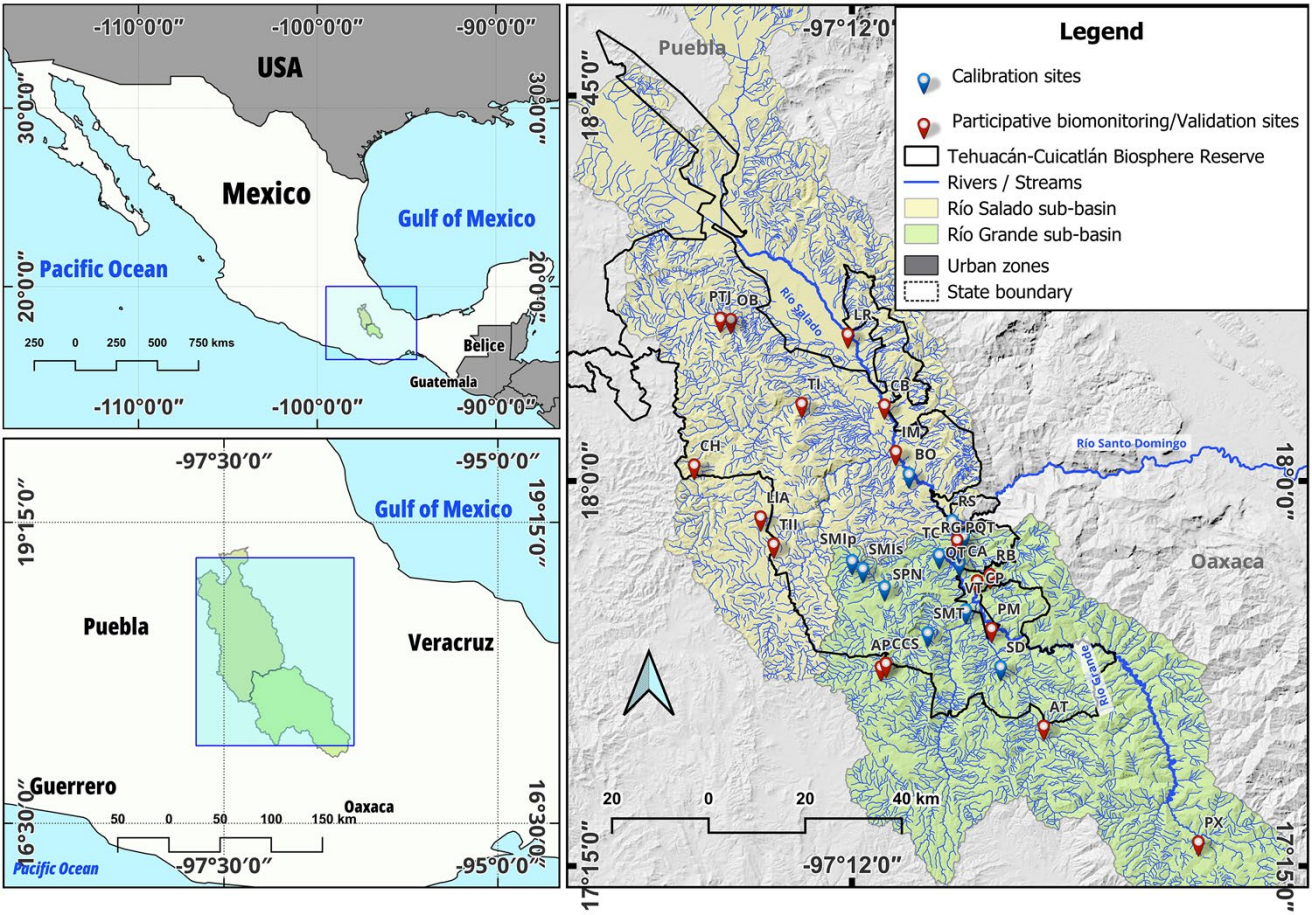
Hydrographic systems in the Tehuacán-Cuicatlán Biosphere Reserve and study sites. The map was generated using the vectorial layers freely available from National Institute of Statistics, Geography and Informatics (https://www.inegi.org.mx/temas/mapadigital), National Commission of Protected Natural Areas (http://sig.conanp.gob.mx/website/pagsig/info_shape.htm). In order to generate the map, all layers underwent processing through the open source software Geographic Information System QGIS 3.34. QGIS is licensed under the General Public License (GNU), which permits the acquisition of its source code through tarballs or the Git repository. The study sites' coordinates were collected using a handheld Monterra^®^ | Garmin GPS device, converted to a digital format, and uploaded as a shapefile.

The Tehuacán-Cuicatlán Biosphere Reserve (TCBR), located in the southeast of Mexico, comprises an area^23^ of 4901.86 km^2^. This reserve is located within two physiographic provinces with unique landscape and climatic features: the Sierra Madre del Sur province, which covers 92.65% of the Natural Protected Area (NPA), and the Trans-Mexican Volcanic Belt to the north, comprising 7.35% of the TCBR area^24^. In hydrological terms, 95% of the TCBR area is part of the Papaloapan River basin, which flows into the Gulf of Mexico. The remaining 5% belongs to the Balsas River basin, which flows into the Pacific Ocean. The Río Salado and Río Grande sub-basins are tributaries of the Papaloapan River (Fig. 1). The Salado River sub-basin occupies 64% of the TCBR area (3137 km^2^) in the northern and central portions of the reserve. Río Salado originates in the arid zone; therefore, it is classified as an endogenous river^12^. The second largest sub-basin in the TCBR is Río Grande, in the southern portion, with 142.15 km^2^ (29% of the TCBR area)^23^. Río Grande originates in the Sierra de Juárez, a mountain range outside the arid zone and the TCBR, and is classified as an exogenous river^12^. The Tehuacán valley (in the Salado River sub-basin) resulted from a series of morpho-tectonic events that gave rise to the mountain systems that delimit it; these systems determine differences in humidity, temperature, precipitation, and evapotranspiration. The TCBR has three types of climate: 73.57% of the reserve is arid and semi-arid; 24.64% is temperate humid and subhumid, and 1.79% is warm humid and subhumid^23^.

### Selection and training of citizens (local inhabitants) to participate in citizen science

The TCBR is administered by the National Commission for Protected Natural Areas (CONANP), an agency of the Federal Government of Mexico. In particular, the TCBR Directorate (DTCBR), which belongs to CONANP, executes the TCBR Management Program that sets out the policies, strategies, and actions to be carried out for the conservation of this reserve. The DTCBR has established participatory monitoring programs for different biological groups (mainly plant species of cacti, birds, and mammals). However, water resources, particularly water quality monitoring, were not included in the participatory monitoring scheme. In this sense, we first approached the DTCBR to describe the potential of biomonitoring focused on aquatic macroinvertebrates based on the Biological Monitoring Working Party (BMWP) index calibrated for the TCBR hydrological systems, following the method of Ruiz-Picos^16^. In addition, we offered training to perform participatory biomonitoring in the TCBR. The agreement from this first approach was that the DTCBR would convene some of the indigenous communities inhabiting the TCBR to be trained in water quality assessment based on bioindication with aquatic macroinvertebrates. In this way, a partnership was formed between authorities, academics, and local citizens to set up a participatory monitoring program in the TCBR. During the workshops, educational material was provided to the participants for the identification of macroinvertebrates (at family level) through photographs. Furthermore, we provided training on the application of BMWP based on the bioindication value of each family following the method of Ruiz-Picos^16^. The collected macroinvertebrate samples were transferred to the Laboratory of Aquatic Ecosystem Health Assessment at the *Instituto Politécnico Nacional,* for taxonomic verification. Field data was collected in field books.

### Aquatic macroinvertebrates

The analyzed data set included two groups: data from the macroinvertebrate community previously recorded by our working group at the following sites (Fig. 1 and Supplementary Table 1): Barranca Oscura (BO) and Río Salado (RS) in the Río Salado sub-basin; and Quiotepec (QT), Santiago Dominguillo (SD), Santa María Texcatitlán (SMT), Valerio Trujano (VT), Tecomavaca (TC), El Cacahuatal (CA), Río Grande (RG), Santa María Ixcatlán Sabinos (SMIs), Santa María Ixcatlán Poza (SMIp), and San Pedro Nodón (SPN) in the Río Grande sub-basin^11^ (Fig. 1). The second group of data included the study sites for participatory biomonitoring with local inhabitants and park rangers. Study sites for participatory biomonitoring were selected in agreement with the DTCBR and the trained local inhabitants, and included the following localities: Casa Blanca (CB), Ignacio Mejía Viejo (IM), El Chacuaco (CH), Las Regaderas (LR), Llano de Agua (LIA), Ojo de Buey (OB), Río Bonete (RB), Tepelmeme (TI), Tepelmeme II (TII), and Paraje del Tío Julio (PTJ) in the Río Salado sub-basin, and Presa Matamba (PM), Apoala (AP), Cascada Cola de Serpiente (CCS), Puente Quiotepec (PQT), Concepción Pápalo (CP), Puente Xia (PX), and Atatlahuaca (AT) in the Río Grande sub-basin (Fig. 1).

Macroinvertebrates were collected using a kick net and a D-net, both with a 500 μm mesh size. Samples were obtained in duplicate using the multihabitat method^25^. In each microhabitat, the collection area was standardized to 1 m^2^ according to Barbour et al.^25^. The collected organisms were fixed in 70% alcohol and transported to the laboratory for sorting and taxonomic identification.

The taxonomic determination of macroinvertebrates was performed with a stereo microscope (Nikon^®^ C-Leds SMZ745T) using specialized identification keys^26–28^ by qualified personnel at the Laboratory of Aquatic Ecosystem Health Assessment at the *Instituto Politécnico Nacional*.

The study sites were visited on at least two occasions. The sites used to calibrate the BMWP correspond to the year 2016 and included the dry (April) and rainy (September) seasons. The study sites were selected considering different land uses, such as the natural vegetation typical of the TCBR and different types of agriculture (see Supplementary Table 1) in both sub-basins (Río Salado and Río Grande). The participatory biomonitoring sites were studied in the dry and rainy seasons (from 2017 to 2019 and from 2021 to 2022). The results are reported as the average of both study periods.

### Water quality analysis

At each study site, the following environmental factors were recorded in situ: atmospheric temperature (°C, with a thermometer included in the EXTECH^®^ anemometer); and water temperature (°C), turbidity (NTU), salinity (PSU), dissolved oxygen (DO mg/L), pH, and conductivity (mS/cm) (using a Quanta^®^ multiparameter probe). Stream flow velocity (m/s) was recorded with a portable HACH^®^ flow meter, and geographic coordinates were recorded with a Sport Trak Maguellan^®^ GPS. At each study site, two 500 mL water samples were collected for water quality assessments, in addition a water sample of 10 mL in Whirl–Pak bags was taken for bacteriological testing. The samples were transported in the dark and refrigerated for laboratory tests.

In the laboratory, water samples were tested for biochemical oxygen demand (BOD_5_ mg/L), chloride (mg/L), alkalinity (mg/L), and total and fecal coliforms (MPN/100 mL), according to APHA^29^. Furthermore, nitrates (mg/L NO_3_), hardness (mg/L), and color (U Pt–Co) were determined according to the Hach DR 3900 spectrophotometer techniques. Laboratory data was recorded in lab books.

### Data analysis

#### Biological monitoring working party index

The BMWP index was calculated for each study site and season using the bioindication values of aquatic macroinvertebrates for the TCBR obtained following Ruiz-Picos et al.^16^ and using the first data set: BO, RS, QT, SD, SMT, VT, TC, CA, RG, SMIs, SMIp, and SPN (Fig. 1). The bioindication values of the BMWP represent the tolerance to organic pollution, ranging from 1 (highly tolerant) to 10 (highly sensitive). The BMWP value of a study site is the sum of the bioindication values for all the families present in it. The BMWP values are represented as the average of the dry and rainy seasons.

#### Water quality index

Physicochemical data recorded in situ and analyzed in the laboratory were used to determine the Water Quality Index (WQI) of Dinius^22^, which is a multiplicative index that includes 11 physicochemical variables and two microbiological variables (atmospheric and water temperature, dissolved oxygen, pH, conductivity, nitrates, alkalinity, hardness, chlorides, true color, BOD_5_, and total and fecal coliforms).

$$WQI = \mathop \prod \limits_{i = 1}^{n} I_{i}^{{w_{i} }}$$

where: *WQI* = Water Quality Index, $\prod\nolimits_{i = 1}^{n} {}$ = represents the operation of multiplying together all terms immediately following it. *li* = subscript of the *i-th* parameter, *Wi* = parameter weighting value, *n* = number of parameters.

The WQI grades six water uses (Public Water Supply, Recreation, Fish, Shellfish, Agriculture, and Industry), assigning different water quality categories according to the use of water. The present work considered only the Public Water Supply and Agriculture uses (Refer to the Supplementary Table 2).

#### Normalized difference vegetation index

The Normalized Difference Vegetation Index (NDVI) was calculated for all study sites in buffer areas established around the stream with zones influenced by land use and land cover, measuring 200 m upstream of the monitoring site by 120 m at both sides (200 m × 120 m). We used this buffer based on our previous studies in the riparian zone of the TCBR^12^ with buffer areas from 50 up to 400 m, and those of Sewneey et al.^30^ and Hill^31^, both suggested 90 m buffers as optimal to achieve the highest possible sediment and nutrient removal efficiency, which is relevant for pollution mitigation in the rivers studied by them. We use 120 m due to the possible presence of low slope areas that could generate larger riparian zones on both bank sides of the stream.

Data were obtained from the free-access sources EO Browser and ladsweb.modaps.eosdis.nasa.gov based on Sentinel 2 imagery for the period of September 2016 to September 2019, and April 2021 to September 2022. All the gathered images met the criteria of 0% to 10% cloudiness. The NDVI is the difference between the reflectance values of bands 8 (near infrared) and 4 (visible–red), divided by the sum of the reflectance values of these two bands:

$$NDVI = \frac{{\left( {NIR - R} \right)}}{{\left( {NIR + R} \right)}}$$

where *NDVI* = Normalized Difference Vegetation Index, *NIR* = Reflectance values of the Near-Infrared band, *R* = Reflectance values of the red band in the visible spectrum.

The mean NDVI values for both seasons studied are represented in bar graphs of the study sites by sub-basins.

#### Geographic expansion test

Additionally, a validation test of the BMWP was carried out using water quality variables from the study sites RB, CH, IM, LlA, AP, PTJ, LR, OB, PQT, and CCS, considering only those variables used as qualifiers in the BMWP calibration process (salinity, conductivity, Cl^-^, NO_2_, NH_3_, color, NO_3_, NT, pH, hardness, SO_4_). With these data, a multiple linear regression was carried out with water quality variables as predictor variables and observed BMWP (BMWP_obs_) as the dependent variable. The resulting equation allowed us to calculate BMWP (BMWP_calc_) values for each site. With the observed and calculated BMW values, a scatterplot was elaborated and a linear regression was obtained with its 95% confidence intervals.

For each index (BMWP, WQI, and NDVI), a data normality test was carried out and the differences between sub-basins and between periods were tested through an ANOVA, previous homoscedasticity test; these analyses were performed with the XLSTAT software ver. 2019. BMWP and WQI values are presented in bar plots with SE.

#### Relationship between BMWP, WQI, and NDVI

An ordination analysis (PCA) of the study sites was carried out considering the BMWP, WQI, and NDVI values as attributes of each study site. The PCA was performed using the Pearson correlation coefficient with the XLSTAT software ver. 2019.

## Results

### Citizen training

The first workshop on the application of participatory biomonitoring was held over 3 days in August 2018 in Tehuacán, Puebla, using the BMWP index as a tool. A total of 36 indigenous participants in the TCBR were trained, including adults, teenagers, and children of both sexes. Academics from local higher-education institutions also attended this workshop to strengthen the academic aspects of the participatory monitoring program. A second 2-day workshop was held in September 2019, training 56 indigenous participants (see Supplementary Table 3, with the names of the localities that participated in the workshops). The adults who participated included municipal authorities. It was agreed that the 92 trained people from 22 local communities would be facilitators to share their knowledge with other members of their communities to increase the number of citizens trained in the application of the BMWP. Once the monitoring program was launched, an increasing number of participants assisted in sampling at different sites of the TCBR hydrological systems, for a total of 143 active persons in participatory monitoring, including the three types of participants: park rangers and municipal authorities (government), researchers and students (academia), and local inhabitants (citizens).

### Citizen science data validation

The family-level identification of specimens collected by the participants and the final BMWP scores were validated in the laboratory by academics using specialized dichotomous keys and the training materials provided to citizen participants. The information produced by academics and through participatory monitoring was analyzed after validation.

### Spatial and temporal variation of the BMWP

**Figure 2:**
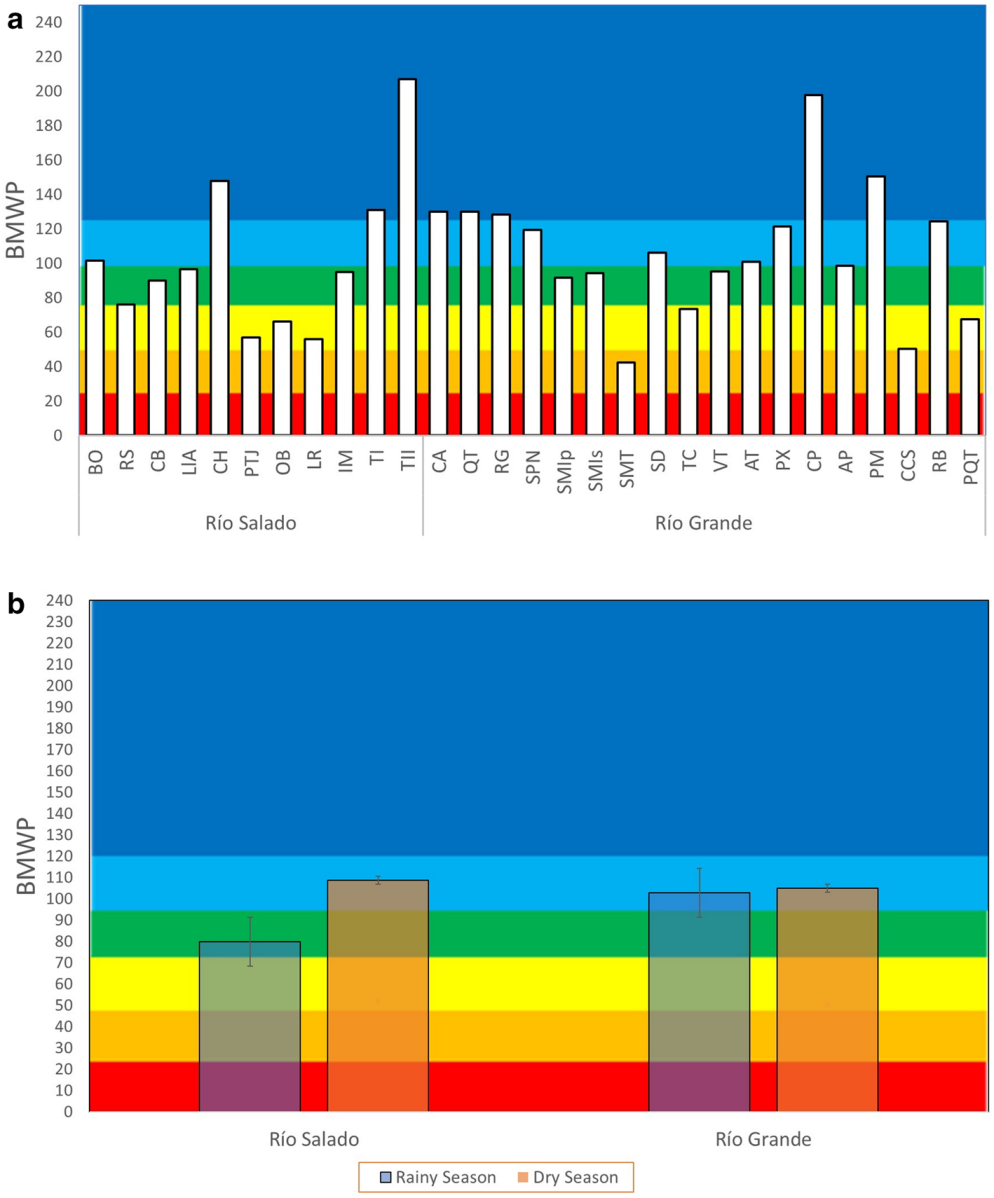
Mean BMWP values. (**a**) For each study site in the Río Salado and Río Grande sub-basins, and (**b**) by season for each sub-basin. Water quality categories: (dark blue) Excellent, (light blue) Very Good, (green) Good, (yellow) Fair, (orange) Poor, (red) Extremely polluted. SE analysis and plot construction were conducted using the Microsoft Excel software from Microsoft Corporation (2018).

The mean BMWP values for each study site in the TCRB (dry and rainy seasons) showed five water quality categories (*Excellent, Very Good, Good, Fair*, and *Poor*). The study sites located in the Río Salado sub-basin recorded water quality categories of *Excellent* (TI, TII, and CH = 27.27%), *Very Good* (BO = 9.09%), *Good* (CB, LIA, and IM = 27.27%), and *Fair* (RS, PTJ, OB, and LR = 36.36%) (Fig. 2a). In the Río Grande sub-basin, the sites reached the categories of *Excellent* (CP, PM, CA, QT, and RG = 27.77%), *Very Good* (SPN, SD, AT, PX, and RB = 27.77%), *Good* (SMIp, SM, VT, AP = 22.22%), *Fair* (TC, CCS, PQT = 16.66%), and *Poor* (SMT = 5.55%) (Fig. 2a).

**Figure 3:**
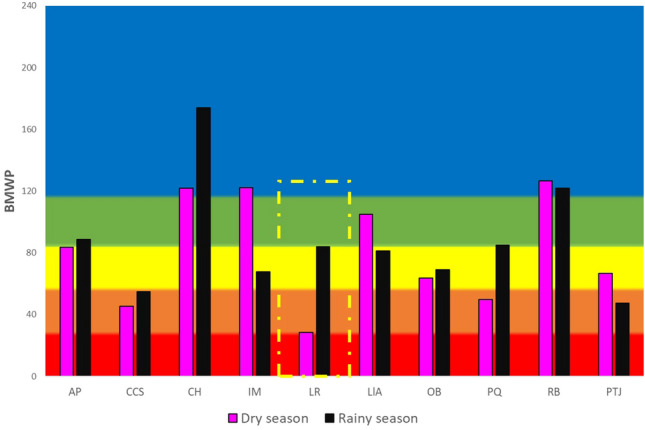
BMWP values by season at the participatory monitoring sites for the Río Salado sub-basin in the dry season of 2021 and the rainy season of 2022. Water quality categories: (dark blue) Excellent, (light blue) Very good, (green) Good, (yellow) Fair, (orange) Poor, (red) Extremely polluted. The plot construction were conducted using the Microsoft Excel software from Microsoft Corporation (2018).

The mean BMWP values by season for stations along the Río Grande sub-basin do not show variations in the water quality category, being *Very Good* ($\overline{X}$_BMWP_ = 104.88 in the dry season and $\overline{X}$_BMWP_ = 102.72 in the rainy season). No significant differences were found between seasons (*p* > 0.05) (Fig. 2b). In the Río Salado sub-basin, the mean water quality category was *Very Good* in the dry season and *Good* in the rainy season ($\overline{X}$_BMWP_ = 108.71 in the dry season and $\overline{X}$_BMWP_ = 79.75 in the rainy season) (Fig. 2b). During participatory biomonitoring, it is worth highlighting the case of the LR study site (Río Salado sub-basin), which showed marked differences between study seasons. In the rainy season (July 2021), the BMWP index was 28.3, which corresponds to the *Poor* water quality category. In the subsequent participatory monitoring season, i.e., the dry season of March 2022, the BMWP increased to 83.8, which corresponds to the *Good* quality category (Fig. 3).

### Spatial and temporal variation in the WQI

**Figure 4:**
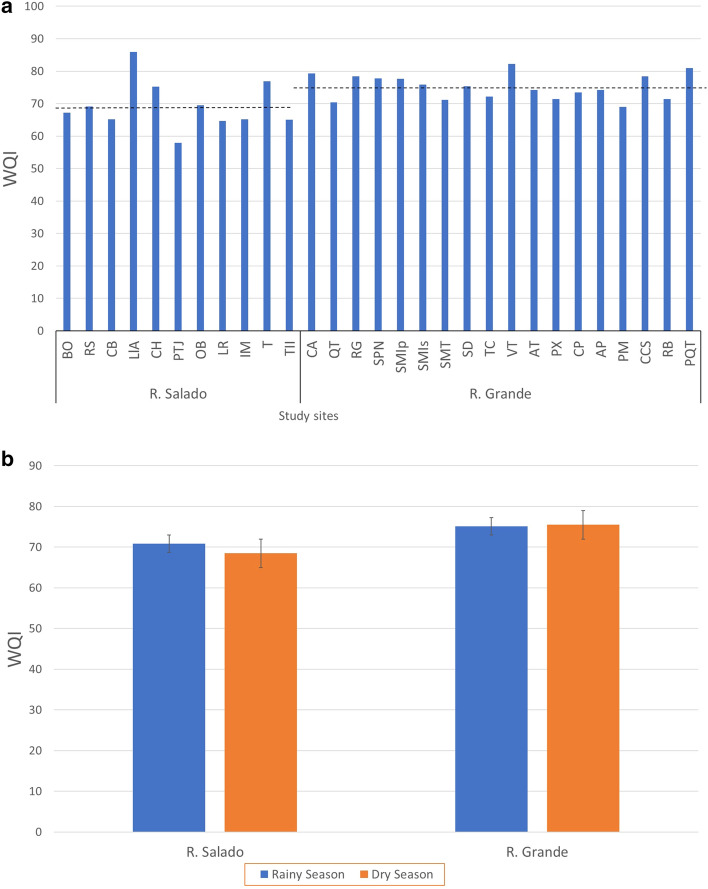
Water Quality Index. (**a**) Mean WQI values (dry and rainy seasons) for the Río Salado and Rio Grande study sites. (**b**) Mean WQI values for the dry and rainy seasons in the Río Salado and Río Grande sub-basins. *p* > 0.05 (Bars indicate the standard error). SE analysis and plots construction were conducted using the Microsoft Excel software from Microsoft Corporation (2018).

In the Río Salado sub-basin, the WQI fluctuated from 57.89 to 85.89; in the Río Grande sub-basin, from 70.40 to 82.18 (Fig. 4a). The mean WQI values in the dry and rainy seasons for the Río Salado sub-basin (69.24 ± 2.29) were lower than those for the Río Grande sub-basin (75.16 ± 1.16); these differences were statistically significant (*p* < 0.05).

The mean WQI per season showed that Río Salado reached a slightly higher value (70.83) in the rainy season, with an *Acceptable* water quality category, than that of the dry season (68.48), with *Mild contamination* category (Fig. 4b).

In the Río Grande sub-basin, the dry and rainy seasons recorded mean WQI values of 75.47 and 75.13 respectively, with an *Acceptable* quality category and with no significant differences between seasons (*p* > 0.05) (Fig. 4b) (see Supplementary Table 4, with WQI for the dry and rainy seasons).

**Figure 5:**
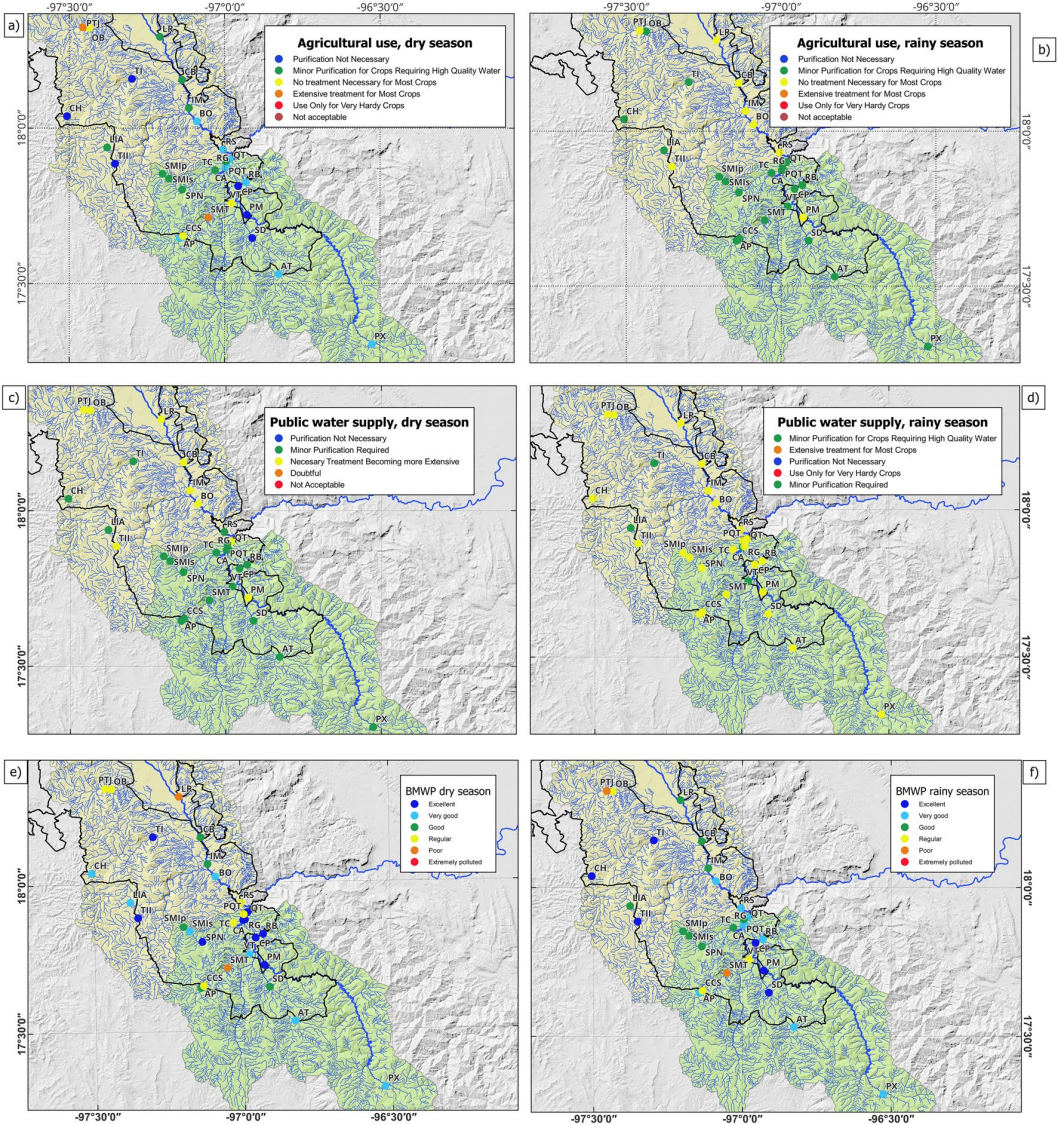
Study sites in the TCRB according to (**a**) WQI related to agricultural use in the dry season, (**b**) WQI related to agricultural use in the rainy season, (**c**) WQI related to human consumption in the dry season, (**d**) WQI related to human consumption in the rainy season (**e**) BMWP in the dry season, and (**f**) BMWP in the rainy season. The regional map was created using vectorial layers that are freely available from the National Institute of Statistics, Geography, and Informatics (https://www.inegi.org.mx/temas/mapadigital) and the National Commission of Protected Natural Areas (http://sig.conanp.gob.mx/website/pagsig/info_shape.htm). All layers were processed using the open-source geographic information system software QGIS 3.34. QGIS is also an open-source software available under the General Public License (GNU), which means that its source code can be downloaded through tarballs or the git repository. The study sites points were downloaded from a hand-held GPS device (Monterra^®^ | Garmin), digitalized, and uploaded as a shapefile. Sampling points and legend layouts were edited using open-source software that is available at https://inkscape.org.

Since there is no industrial activity in the TCBR, the WQI results were analyzed considering the use of water for agriculture (mainly) and human consumption. Considering mean scores and regarding agricultural use, 73% of the study sites in the Río Salado sub-basin were given the category of *No Treatment Necessary for Most Crops* (BO, RS, CB, PTJ, OB, LR, IM, TII). The remaining 27% corresponded to the category of *Minor Purification for Crops Requiring High-Quality Water* (LlA, CH, TI). In the Río Grande sub-basin, 95% of the study sites received the category of *Minor Purification for Crops Requiring High-Quality Water* (CA, QT, RG, SPN, SMip, SMis, SMT, SD, TC, VT, AT, PX, CP, AP, CCS, RB, PQT), and a single site corresponded to the category of *No Treatment Necessary for Most Crops* (PM), representing 5% of all sites sampled in this sub-basin (Fig. 5a).

Regarding the use of water for human consumption and considering mean scores, 91% of the study sites in the Río Salado sub-basin corresponded to the category of *Necessary Treatment Becoming More Extensive* (BO, RS, CB, CH, PTJ, OB, LR, IM, TI, TII). The remaining 9%, representing a single study site, was assigned the category of *Minor Purification Required* (LlA). In the Río Grande sub-basin, 89% of the study sites were assigned the category of *Necessary Treatment Becoming More Extensive* (CA, QT, RG, SPN, SMIp, SMIs, SMT, SD, TC, AT, PX, CP, AP, PM, CCS, RB). The remaining two study sites, corresponding to 11% (VT, PQT) were assigned the *Minor Purification Required* category (Fig. 5b).

### Spatial and seasonal distribution of the water quality index and the biological monitoring working party

In the Río Salado sub-basin, The WQI returned only two categories, regardless of the use of water for irrigation (*No Treatment Necessary for Most Crops* and *Regular and Minor Purification for Crops Requiring High-Water Quality*) or for human consumption (*Necessary Treatment Becoming More Extensive* and *Minor Purification Required*) (Fig. 5a,b).

In the Río Salado sub-basin during the dry season, the WQI categories for the use of water for irrigation, ranged from Extensive treatment for most crops (PTJ) to Purification no necessary (CH, TI, and TII) (Fig. 5a), while in the rainy season only two categories were presented (No Treatment Necessary for Most Crops and Regular and Minor Purification for Crops Requiring High-Water Quality) (Fig. 5b). The use for human consumption shows only two categories in both seasons and both sub-basins: Necessary Treatment Becoming More Extensive and Minor Purification Required (Fig. 5c,d). The bioindication-based evaluation (BMWP) differentiates more water quality categories, assigns *Excellent* and *Very Good* water quality to study sites associated with the upper portion of the basins, and differentiates study sites with *Fair* and *Poor* water quality (Fig. 5e,f). In sites with low BMWP values, the persons in charge of participatory monitoring identified the main issues affecting water quality, pointing out livestock activities, open dumps, and small runoff from upstream town discharges, among others.

### BMWP validation and geographic extension test

The validation process to obtain the value of BMWP_calc_ from the environmental variables returned a coefficient of determination *R*^2^ of 0.519 (calculated *vs* observed), and the following equation:

$${\text{BMWP}}_{{{\text{calc}}}} = \, \left( {92.697 \, - \, 5.982 \, *{\text{ N}}\;{\text{total }} + \, 260.634 \, *{\text{ NO}}_{2} } \right)$$

**Figure 6:**
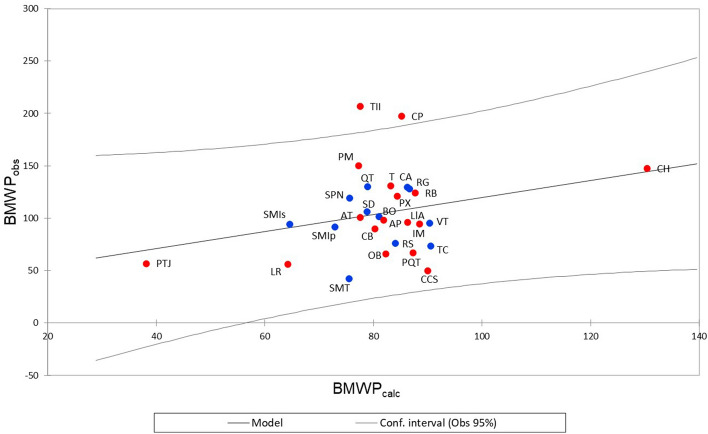
Linear regression of BMWP (observed vs. calculated). Blue dots are study sites for BMWP calibration; red dots are study sites included in the participatory monitoring. The statistical software XLSTAT was used to create the current plot under licensed Lumivero (2019).

The scatterplot of BMWP_calculated_ and BMW_observed_ values from the participatory monitoring study sites (RB, CH, IM, LlA, AP, PTJ, LR, OB, PQT, CCS, CB, AT, PX, T, PM) showed that all study sites lie within the 95% confidence interval (Fig. 6), except for the Concepción Pápalo (CP) and Tepelmeme II (TII) sites. These two sites are located at the headwaters of the Rio Grande tributaries with little human intervention; this explains why these sites reached the highest BMWP values.

Since participatory monitoring sites were not included in the BMWP calibration, these sites are considered external to the model. In this way, by using the model with the results of participatory monitoring and finding that the points fall within its 95% confidence interval, it is demonstrated that the model fits the participatory monitoring sites. As these sites comprise a larger geographic area than that used for calibration, the use of the BMWP index is validated over a larger geographic area. Therefore, the BMWP index can be used with confidence in a larger area in the TCBR, with no recalibration required at this time.

Similarly, the study site that recorded the lowest observed BMWP values was Santa María Texcatitlán (SMT), the former site and Cascada Cola de Serpiente CCS are study sites that need management to improve water quality.

### Normalized difference vegetation index

**Figure 7:**
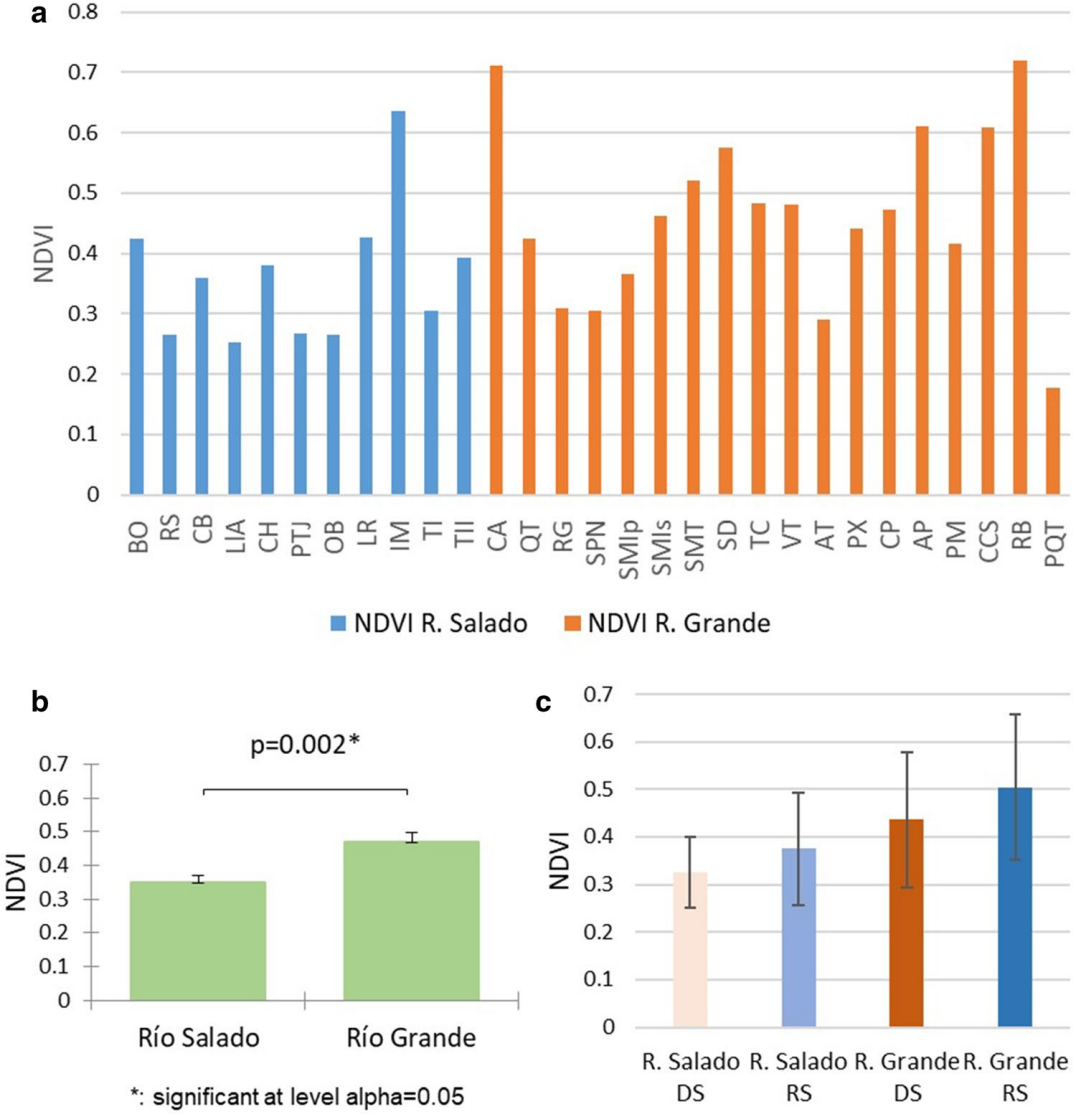
NDVI values for study sites in the Río Salado and Río Grande sub-basins. (**a**) Mean NDVI values for each study site (dry and rainy seasons), (**b**) Overall mean NDVI values for the Río Salado and Rio Grande sub-basins, (**c**) Mean NDVI values sorted according to the dry and rainy seasons for each sub-basin, Río Salado and Río Grande. SE analysis and plots construction were conducted using the Microsoft Excel software from Microsoft Corporation (2018). The statistical analysis was performed using the statistical software XLSTAT under licensed Lumivero (2019).

The mean NDVI values (dry and rainy seasons) for the study sites of the Salado River sub-basin ranged from 0.20 to 0.63 with an overall mean of 0.36 (± an SE of 0.03), while those of Río Grande ranged from 0.6 to 0.74, with an overall mean of 0.46 (± 0.007) (Fig. 7a). Significant differences in NDVI were observed between Río Salado and Río Grande (*p* > 0.05) (Fig. 7b).

The analysis by season (dry and rainy seasons) reveals higher NDVI values in the rainy season in both sub-basins, with the highest value in Río Grande ($\overline{X}$ = 0.74 ± 0.11 in Río Grande and $\overline{X}$ = 0.633 ± 0.07 in Río Salado); in the dry season, the lowest values were recorded in Río Salado ($\overline{X}$ = 0.20 ± 0.074) (Fig. 7c).

### Index integration (BMWP, WQI, and NDVI)

**Figure 8:**
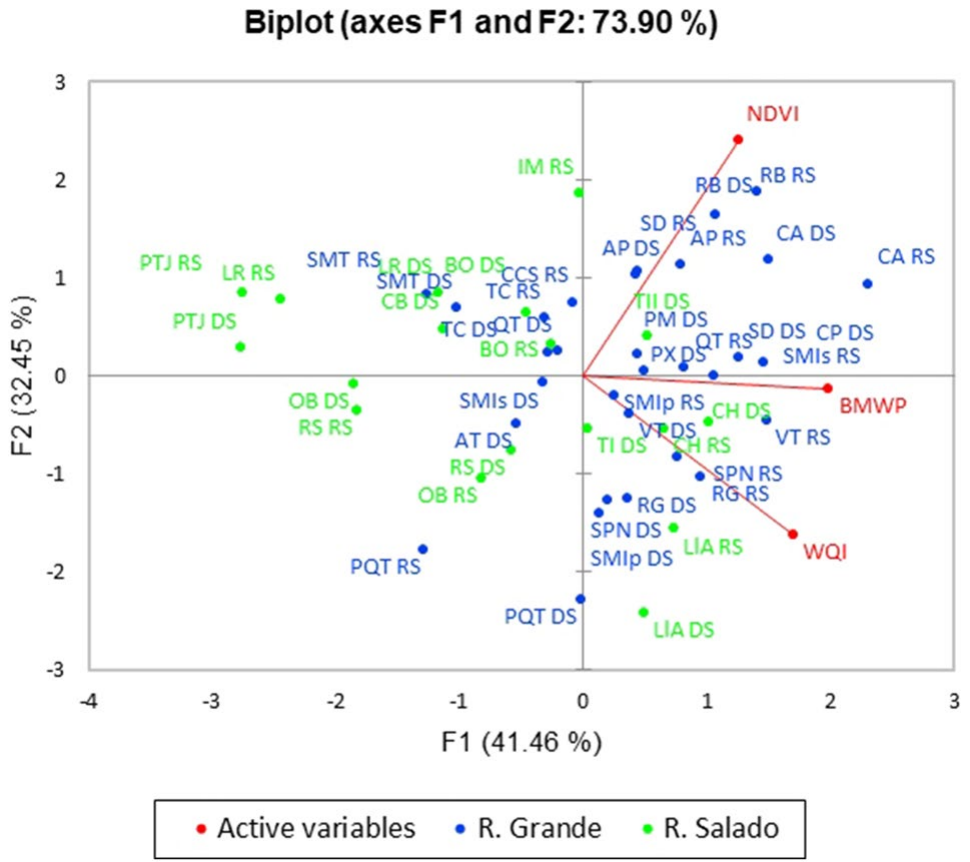
Ordination of TCBR study sites in both study seasons according to their BMWP, WQI and NDVI values. Letters after the study site acronym represent the dry season (DS) and the rainy season (RS). The analysis and plot was performed and created using the statistical software XLSTAT under licensed Lumivero (2019).

The PCA of the study sites according to the BMWP, WQI, and NDVI indices yielded 73.90% in the first two principal components (PC1 and PC2). The biplot shows the ordination of study sites according to a gradient of BMWP values in the first component (F1) (Fig. 8). The study sites that obtained high BMWP values are mostly from the Río Grande sub-basin; additionally, some sites located in the Río Salado sub-basin, such as CH, LlA (rainy season) and TII, CH, TI, and LlA (dry season), also achieved high BMWP values. BMWP showed a close association with WQI, indicating that BMWP is an excellent predictor of physicochemical water quality. The second component (F2) showed the ordination of study sites along a gradient of NDVI values. In this component, the sites with high NDVI values are located mainly in the Río Grande sub-basin, in addition to site IM RS of the Río Salado sub-basin. The lowest NDVI values were recorded mainly in Río Salado, along with some sites located in Río Grande (PQT, RS, and DS).

## Discussion

Biomonitoring in the TCBR using the BMWP index provided an overall view of the gradient of water quality conditions in the rivers of this reserve. In general, the mean BMWP values corresponded to the *Good* water quality category; however, despite their location within a biosphere reserve, four study sites in Río Salado and three in Río Grande (36% of study sites in Río Salado and 22% in Río Grande) showed water quality corresponding to the *Fair* category, and one study site in Río Grande was given the *Poor* water quality category. Although no significant differences were detected between seasons (dry and rainy), lower BMWP values were observed during the rainy season, with a more pronounced difference in Río Salado than in Río Grande. During the rains, runoff over the basin incorporates different compounds to the river associated with the land uses in the basin^32^ and related to its geological nature. These compounds may contribute to reducing water quality, which was reflected in the BMWP. However, the differences between seasons were of low magnitude, reflecting a minor impact from the catchment basin. For its part, the overall *Good* water quality highlights the favorable condition of the water bodies in the TCBR, as well as the effectiveness of the management plan in the reserve. In this sense, Bona et al.^33^ used aquatic macroinvertebrates (through the BMWP) and diatoms as bioindicators of the condition of lotic systems in one of the oldest reserves in the heart of the western Alps in Italy. These authors reported the effectiveness of natural areas to preserve the favorable state of running water ecosystems and concluded that limiting hydrological alterations increases the resilience of aquatic communities.

Participatory monitoring in the TCBR included three stakeholders: decision makers, local inhabitants, and academics, who participated in the key biomonitoring activities: the DTCBR (decision-makers) convened local communities, citizens (local inhabitants) received training and participated in the monitoring, and collaborating researchers (academics) who provided training through workshops and assistance during the monitoring, along with TCBR park rangers. This triple partnership made it possible to reach agreements on the study sites to be monitored, and on the groups of citizens who participated in the training workshops and the monitoring of water bodies adjacent to their communities. In this way, information was collected to assess the water quality condition covering a large area of the TCBR. According to Costa et al.^34^, participatory monitoring represents an innovative approach to managing biodiversity conservation in protected natural areas, in addition to being less expensive than the conventional evaluation of physicochemical characteristics. For example, all the reagents involved in the physicochemical testing of water quality, the laboratory equipment necessary to carry out these tests (spectrophotometers, hot plate, and coolers, among others), and laboratory glassware are far more expensive than a kicking net, alcohol for preserving the samples, and a stereomicroscope. This study showed that participatory monitoring is not only useful for the conservation of biodiversity, but is particularly relevant in the management of water bodies. In terms of resource management, the results of biomonitoring have allowed not only the water quality assessment but also supported decision-making, as it builds the community capacities of native peoples. They actively promoted the use of information to implement sanitation actions in collaboration with municipal authorities and disseminated information in focus groups, in addition to promoting actions to care for their environment. This highlights the sense of belonging and conservation of the water resources that TCBR residents have acquired. During our monitoring, we detected a study site, “Las Regaderas LR” (Salado River sub-basin), where the BMWP results were very low, corresponding to the *Poor* water quality category (28.3 in July 2021), associated with the wastewater discharges from a nearby town (Zinacatepec, Puebla). These findings were shared with the municipal authorities, who took steps to avoid the discharge of wastewater into the river to conserve and protect the water resource. Thus, the following participatory monitoring in the dry season (March 2022) recorded the recovery of the river with a BMWP value of 83.8, corresponding to the *Good* water quality category. These results show the high sensitivity of the BMWP index^16^ and the recovery capacity of the TCBR river ecosystems after disturbances. In addition, these findings highlight the resilience of this section of the river. The study site “Cascada Cola de Serpiente CCS” has a Poor category and currently, the municipal authorities are involved to reactivate the operation of a wastewater treatment plant in the town and thus improve the quality of the water at this study site.

On the other hand, according to English et al.^35^, the so-called “Pyramid of participatory research approaches” involves three levels of public participation. At the base of the pyramid, citizens only focus on data collection; at the second level, citizens also participate in defining the issue; last, at the apex of the pyramid, the so-called “extreme” participatory monitoring considers that citizens engage in the analysis and interpretation of the data, and participate in the actions taken. This last level is desirable in all participatory monitoring. In the TCBR, the “Las Regaderas” study site resulted in reaching the top of the pyramid because the detection of *Poor* water quality at that site prompted citizens to take actions to recover the river. This process led to the success of the participatory biomonitoring of the TCBR where local inhabitants not only assisted in the monitoring process and learned to evaluate water quality, but also participated in management by promoting its recovery and, consequently, prevented any further deterioration in that section of the river. The study site Cascada Cola de Serpiente is considered in a program to improve its water quality.

The WQI showed that the physicochemical water quality is highly homogeneous within each sub-basin, with the mean WQI values for Río Salado (69.24 ± 2.29 SE) being lower than those for Río Grande (75.16 ± 1.16 SE). The low WQI values for the sites in the Salado River sub-basin are related to the geological nature of the basin. The high chloride, conductivity, and hardness levels (all of which lower the WQI value) are typical of the geological stratum. The Salado River sub-basin has sedimentary rocks from the Lower Tertiary that include sandstones and conglomerates, shales, limestone rocks, limonite, and gypsum, as well as Cretaceous sedimentary rocks such as limestone, volcano-sedimentary rocks, shales, sandstones, and conglomerates^13^. These geological characteristics suggest that this region was submerged in the past and, currently, these contribute to hard and salty waters^11^, hence the name of “Río Salado” (salty river). This peculiarity allows the exploitation of salt in this river^13^. An increase in WQI was observed in Río Salado during the rainy season, which can be attributed to the dilution of salts in the river. This dilution process has been recorded in other salty rivers such as the Athi River, Kenya^36^. In Río Grande, a non-salty river, the WQI dropped slightly during the rains as a result of runoff from the basin that contributed materials to river water, as also reported for rivers in the Mun River basin in Thailand^37^.

Several authors have identified that changes in land use and land cover have influenced the quality of water bodies in affected watersheds^38,39^. One of the indices that allows characterizing land use and occupation in watersheds is the Normalized Difference Vegetation Index (NDVI), which is a function of the red and near-infrared spectral bands. The NDVI is useful to evaluate vegetation health^36^ and to establishing the relationships between land use and land cover with water quality^40^. NDVI values range between − 1 and 1. The NDVI provides information about the vigor and photosynthetic capacity of the vegetation canopy^41^. Thus, the principle of NDVI is to measure the greenness intensity, which is correlated with vegetation density^42^. In this way, the higher the NDVI value, the higher the vegetation density, and vice versa, only applicable for positive values^43,44^. In this study, we used the NDVI for riparian zones due to the relevant role of this zone in regulating inputs of nutrients, organic matter, and even xenobiotics^45^. These multiple stressors can exert cumulative effects that could produce cascading effects on biodiversity and ecosystem functioning^46^. Thus, the riparian zone is an important target for stressor-mitigation or biomonitoring actions^47^. In arid areas, the NDVI is usually between 0 and 0.2 because these areas are usually characterized by rock or bare soil. The TCBR is located in an arid semi-arid area with the typical vegetation of these regions (columnar cactus forests, low deciduous forests, and pine-oak forests in highland areas). Maldonado-Enriquez et al.^48^ reported low NDVI values for arid areas in northwest Mexico. Sedeño-Díaz and López-López^12^ evaluated the NDVI of the Río Salado and Río Grande sub-basins and their respective riparian corridors in the TCBR during the dry and rainy seasons. These authors recorded NDVI values from 0.45 (dry season) to 0.56 (rainy season) in the Río Grande sub-basin and from 0.43 (dry season) to 0.55 (rainy season) in the Río Grande corridor. In the case of the Río Salado, the NDVI values varied from 0.28 (dry season) to 0.43 (rainy season) in the sub-basin and from 0.28 (dry season) to 0.31 (rainy season) in the corridor. These values are consistent with the values recorded in the present study, where the NDVI was lower for the study sites in the Río Salado sub-basin. We used the NDVI considering that riparian vegetation should be able to improve the water quality in several ways: including acting as a sediment sink, filtering stormwater runoff, retaining soil, fostering habitat heterogeneity, and removing nutrients from water^12^. The PCA showed that despite the low NDVI values, the sites that reached the highest values also have high BMWP values. This means that, although vegetation cover is sparse, areas with high NDVI favor a better condition in water bodies that support aquatic macroinvertebrate communities, associated with high BMWP values. In contrast, sites with lower NDVI values also showed lower BMWP. In the present study, sites in the Rio Grande sub-basin were associated with high NDVI values, while sites in the Río Salado sub-basin had lower NDVI values. The above also agrees with the findings reported by Sedeño-Díaz and López-López^12^, who pointed out that the fluvial corridors of the endogenous rivers (Río Salado sub-basin) are subjected to more drastic processes and conditions than exogenous fluvial corridors (Río Grande sub-basin).

The enormous positive influence of riparian vegetation in protecting water bodies from the effects of human activity in the catchment basin has been noted^49^, particularly in areas with agricultural activity^39^. The deterioration of riparian zones promotes an increase in riverbank erosion, increasing the influx of sediments, and reducing the capacity to filter pollutants from land runoff^50^. In this sense, protecting riparian vegetation in the TCBR is highly relevant for promoting *Good* water quality in the river systems.

Considering the factors that lead to lower WQI values in the Río Salado sub-basin and the differences in BMWP and NDVI between the sub-basins, the Río Salado sub-basin is more susceptible to desertification processes and climate change, as pointed out by Sedeño-Díaz and López-López^12^, with the consequent greater deterioration in water quality. The continued participatory monitoring by the local inhabitants will allow them to detect changes in water quality in both sub-basins and contribute to the development and implementation of actions to restore any adverse trend. Being the study area a natural protected area, the leading instrument of the TCBR is the Management Program^13^. Under the Knowledge subprogram, this instrument includes the strategy to define the priority lines of research and monitoring and sets the bases of collaboration with academic institutions and universities regarding the production, documentation, and systematization of information. Based on the above, the Directorate of the TCBR has incorporated the research and monitoring of water bodies through the BMWP methodology in the Annual Operating Program (POA)—the budgetary instrument that defines the annual planning of activities. In this way, the Directorate Reserve Management ensures continued participatory water quality monitoring within the TCBR, allocating resources on an annual basis in which park rangers and native peoples participate with the academic support of our working group.

## Conclusions

In the present study, BMWP was a suitable tool to evaluate water quality in rivers flowing through the TCBR, with a higher sensitivity and more cost-effective than WQI. Participatory monitoring in this reserve began with the training of the local inhabitants and advanced to the top of the pyramid of participatory research approaches, which was successful in managing the recovery of a site previously affected by wastewater discharges. In the TCBR, participatory monitoring contributed to scientific knowledge, which led to the empowerment of indigenous inhabitants to implement actions seeking the recovery of the most affected site. This case showed that the rivers in the TCBR have a high potential for recovery and resilience. In general, according to the BMWP, the water quality of rivers flowing through the TCBR is *Good*, demonstrating the efficiency of the management and conservation plan carried out in this reserve. The PCA showed that the WQI is strongly related to the BMWP; however, the BMWP was much more sensitive in detecting areas affected by domestic wastewater discharges. In general, NDVI values were low in the TCBR, being lower in the Río Salado sub-basin, and did not show a close relationship with BMWP values. However, areas with higher NDVI values generally showed higher BMWP values. The results of the present study highlight the usefulness of biomonitoring and the contribution of local inhabitants to monitor water quality in biosphere reserves and implement timely measures to keep rivers in good condition. The participatory monitoring described here succeeded in converting the TCBR from an area lacking scientific knowledge on water quality to a natural protected area with in-depth spatial and temporal monitoring of water quality supported by bioindication.

### Supplementary Information

Supplementary Table 1.

Supplementary Table 2.

Supplementary Table 3.

Supplementary Table 4.

## Data Availability

The datasets used and/or analyzed during the current study are available from the corresponding author upon reasonable request.
