# Supplementary material for: Indigenous people doing citizen science to assess water quality using the BMWP in rivers of an arid semi-arid biosphere reserve in Mexico
Source: Sci Rep. 2024 Jul 2;14:15090. doi: 10.1038/s41598-024-65903-7 (PMC11219914; doi:10.1038/s41598-024-65903-7)
Supplement: Supplementary file 1 — Supplementary Table 1. [file 41598_2024_65903_MOESM1_ESM.docx]

Supplementary Table 1. Study sites, coordinates, and description of land use and land cover.

| **Sub basin** | **Study site** | **Abreviature** | **Latitude** | **Longitude** | **Land Use, Land Cover** |
| --- | --- | --- | --- | --- | --- |
| Rio Grande | Apoala | AP | 17.65 | -97.14 | Induced grassland and Oak Forest |
|  | Atlatlahuca | AT | 17.53 | -96.83 | Irrigated agriculture and Low deciduous forest |
|  | Cacahuatal | CA | 17.87 | -97.00 | Low deciduous forest |
|  | Cascada Cola de Serpiente | CCS | 17.65 | -97.13 | Seasonal agriculture and Low deciduous forest |
|  | Concepción Pápalo | CP | 17.81 | -96.96 | Low deciduous forest |
|  | Malambo "Presa" | PM | 17.72 | -96.93 | Low deciduous forest and irrigated agriculture |
|  | Puente Quiotepec | PQT | 17.89 | -97.00 | Irrigated agriculture |
|  | Puente Xia | PX | 17.31 | -96.53 | Oak forest |
|  | Quiotepec | QT | 17.90 | -96.98 | deciduous forest and Human settlements |
|  | Río Bonete | RB | 17.83 | -96.93 | Low deciduous forest |
|  | Río Grande | RG | 17.88 | -96.99 | Low deciduous forest |
|  | Santiago Dominguillo | SD | 17.65 | -96.91 | Low deciduous forest |
|  | Santa María Ixcatlán poza | SMIp | 17.85 | -97.20 | Seasonal agriculture |
|  | Santa María Ixcatlán Sabinos | SMIs | 17.84 | -97.18 | Seasonal agriculture |
|  | Santa María Texcatitlán | SMT | 17.71 | -97.05 | Irrigated agriculture |
|  | San Pedro Nodon | SPN | 17.80 | -97.14 | Seasonal agriculture |
|  | Tecomavaca | TC | 17.86 | -97.03 | Low deciduous forest |
|  | Valerio Trujano | VT | 17.76 | -96.98 | Low deciduous forest |
| Rio Salado | Barranca Obscura | BO | 18.06 | -97.12 | Low deciduous forest |
|  | Casa Blanca | CB | 18.16 | -97.14 | Irrigated agriculture |
|  | El Chacuaco | CH | 18.04 | -97.51 | Tascate Forest |
|  | Ignacio Mejía Viejo | IM | 18.06 | -97.11 | Low deciduous forest |
|  | Llano de Agua | LlA | 17.94 | -97.38 | Induced grassland |
|  | Las Regaderas | LR | 18.29 | -97.21 | Irrigated agriculture |
|  | Ojo de Buey | OB | 18.32 | -97.45 | Seasonal agriculture |
|  | Paraje Tío Julio | PTJ | 18.32 | -97.46 | Seasonal agriculture |
|  | Río Salado | RS | 17.93 | -97.01 | Irrigated agriculture and Low deciduous forest |
|  | Tepelmeme | TI | 18.16 | -97.30 | Low deciduous forest |
|  | Tepelmeme II | TII | 17.89 | -97.35 | Induced grassland |
