# Supplementary material for: Indigenous people doing citizen science to assess water quality using the BMWP in rivers of an arid semi-arid biosphere reserve in Mexico
Source: Sci Rep. 2024 Jul 2;14:15090. doi: 10.1038/s41598-024-65903-7 (PMC11219914; doi:10.1038/s41598-024-65903-7)
Supplement: Supplementary file 2 — Supplementary Table 2. [file 41598_2024_65903_MOESM2_ESM.docx]

Supplementary Table 2. General rating scale for water use (taken from Dinius 1987)

| **Level of Pollution (100 = Best)** | **Water Uses** | |
| --- | --- | --- |
|  | **Public Water Supply** | **Agricultural** |
| 100 | Purification Not Necessary | Purification Not Necessary |
| 90 | Minor Purification Required | Minor Purification for Crops Requiring High Quality  Water |
| 80 | Necesary Treatment Becoming more Extensive |  |
| 70 |  |  |
|  |  | No treatment Necessary for Most Crops |
| 60 |  |  |
| 50 | Doubtful | Extensive treatment for Most Crops |
| 40 | Not Acceptable |  |
| 30 |  | Use Only for Very Hardy Crops |
| 20 |  | Not Acceptable |
| 10  0 |  |  |
