# Supplementary material for: Indigenous people doing citizen science to assess water quality using the BMWP in rivers of an arid semi-arid biosphere reserve in Mexico
Source: Sci Rep. 2024 Jul 2;14:15090. doi: 10.1038/s41598-024-65903-7 (PMC11219914; doi:10.1038/s41598-024-65903-7)
Supplement: Supplementary file 3 — Supplementary Table 3. [file 41598_2024_65903_MOESM3_ESM.docx]

| **Supplementary** **Table 3.** Name of indigenous communities trained in participatory monitoring | | | |
| --- | --- | --- | --- |
| **Province** | **Municipality** | **Community** | **Sub basin** |
| Oaxaca | Concepción Pápalo | Concepción Papalo | Río Grande |
|  | San Juan Baustista Cuicatlán | San Juan Baustista Cuicatlán | Río Grande |
|  | San Juan Bautista Atatlahuca | San Juan Bautista Atatlahuca | Río Grande |
|  | San Juan Bautista Cuicatlán | Concepción Pápalo | Río Grande |
|  | San Juan Bautista Cuicatlán | San Juan Bautista Cuicatlán | Río Grande |
|  | San Juan Bautista Cuicatlán | Santiago Quiotepec | Río Grande |
|  | San Juan Chicomezúchil | San Juan Chicomezúchil | Río Grande |
|  | Santiago Apoala | Santiago Apoala | Río Grande |
|  | Santiago Nacaltepec | Nacaltepec | Río Grande |
|  | San Antonio Nanahuatípan | Casa Blanca | Río Salado |
|  | Santa María Tecomavaca | Santa María Tecomavaca | Río Salado |
|  | Teotitlán de Flores Magón | Ignacio Mejía | Río Salado |
|  | Teotitlán de Flores Magón | Teotitlán de Flores Magón | Río Salado |
|  | Tepelmeme Villa de Morelos | Rancho de Rogelio | Río Salado |
|  | Tepelmeme Villa de Morelos | Tepelmeme Villa de Morelos | Río Salado |
| Puebla | Caltepec | San Simón Tlacuilotepec | Río Salado |
|  | Caltepec | San Luis Atolotitlán | Río Salado |
|  | Caltepec | Caltepec | Río Salado |
|  | Caltepec | Santiago Coatepec | Río Salado |
|  | Caltepec | Acatitlán | Río Salado |
|  | San José Miahuatlán | San José Axusco | Río Salado |
|  | Tehuacán | Sierra Negra | Río Salado |
|  | Tehuacán | San Pablo Tepetzingo | Río Salado |
|  | Zapotitlán | Ojo de Buey | Río Salado |
|  | Zapotitlán | Salinas del Tio Julio | Río Salado |
|  | Zapotitlán | Los Reyes Metzontla | Río Salado |
|  | Zapotitlán | Zapotitlán Salinas | Río Salado |
|  | Zapotitlán | San Martín del Valle | Río Salado |
|  | Zapotitlán | Santa Teresa | Río Salado |
|  | Zinacatepec | San Sebastian Zinacatepec | Río Salado |
