# Supplementary material for: Indigenous people doing citizen science to assess water quality using the BMWP in rivers of an arid semi-arid biosphere reserve in Mexico
Source: Sci Rep. 2024 Jul 2;14:15090. doi: 10.1038/s41598-024-65903-7 (PMC11219914; doi:10.1038/s41598-024-65903-7)
Supplement: Supplementary file 4 — Supplementary Table 4. [file 41598_2024_65903_MOESM4_ESM.docx]

**Supplementary Table 4.** WQI for each study site in the Dry and Rainy seasons.

| Basin | Study site | Mean Dry Season | Mean Rainy Season |
| --- | --- | --- | --- |
| R. Salado | BO | 66.07 | 68.25 |
|  | RS | 70.75 | 67.43 |
|  | CB | 63.35 | 66.92 |
|  | LlA | 88.15 | 83.65 |
|  | CH | 74.16 | 76.33 |
|  | PTJ | 60.46 | 55.32 |
|  | OB | 63.52 | 75.57 |
|  | LR | 62.60 | 66.61 |
|  | IM | 65.01 | 65.19 |
|  | TI | 70.70 | 83.07 |
|  | TII | 65.09 | 65.09 |
| R. Grande | CA | 78.95 | 79.55 |
|  | QT | 69.18 | 71.62 |
|  | RG | 78.94 | 78.00 |
|  | SPN | 79.33 | 76.17 |
|  | SMIp | 79.46 | 75.88 |
|  | SMIs | 73.65 | 77.99 |
|  | SMT | 71.82 | 70.51 |
|  | SD | 77.34 | 73.29 |
|  | TC | 70.48 | 73.75 |
|  | VT | 81.47 | 82.91 |
|  | AT | 69.61 | 75.27 |
|  | PX | 72.81 | 70.10 |
|  | CP | 73.41 | 73.41 |
|  | AP | 69.61 | 75.27 |
|  | PM | 68.95 | 68.95 |
|  | CCS | 77.13 | 77.13 |
|  | RB | 70.78 | 71.93 |
|  | PQT | 83.54 | 78.31 |
